# Supplementary material for: Urea Detection in Phosphate Buffer and Artificial Urine: A Simplified Kinetic Model of a pH-Sensitive EISCAP Urea Biosensor
Source: Sensors (Basel). 2025 Oct 26;25(21):6596. doi: 10.3390/s25216596 (PMC12608829; doi:10.3390/s25216596)
Supplement: Supplementary file 1 [file sensors-25-06596-s001.zip › Supplementary InformationPoster.pdf]

Supplementary Information S1

Quantitative Analysis of a pH-Sensitive EISCAP Urea Biosensor Using a Simplified Kinetic Model: Parameter Estimation and Matrix Effects

K. Simonyan[1], A. Tsokolakyan[1], V. Buniatyan[1], A. Badasyan*[2] M. Yeranosyan[1,3]

[1] Innovation Centre for Nanoscience and Technologies, A.B. Nalbandyan Institute of Chemical Physics NAS RA, P. Sevak 5/2, 0014 Yerevan, Armenia

[2] Materials Research Laboratory, University of Nova Gorica, Vipavska 13, 5000 Nova Gorica, Slovenia

[3] Institute of Physics, Yerevan State University, A. Manoogian 1, 0025 Yerevan, Armenia

The results presented in this work are part of the research project "UroLogicChip device" supported by the Higher Education and Science Committee of MESCS RA (Research project № 22rl-056). The concept of the „UroLogicChip“ and first results related to urea-sensitive EISCAPs were presented at the 15th International Workshop on Engineering of Functional Interfaces (EnFI 2024, August 29-30, 2024, Linz, Austria) (see Poster below).

Poster S1

Detection of urea in artificial urine using capacitive field-effect biosensors modified with a stacked polyelectrolyte-enzyme bilayer

National Academy of Sciences A.B. Nalbandyan Institute of Chemical Physics

A. Tsokolakyan[1], Ts. Poghosyan[1], V. A. Hayrapetyan[1], D. Petrosyan[1], K. Simonyan[1], A. Badasyan[2], V. Buniatyan[1], T. Karschuck[3], M. Welden[3], H. Iken[3], M. J. Schöning[3], M. Yeranosyan[1], A. Poghossian[4]

astghik.tsokolakyan@edu.isec.am

[1]A.B. Nalbandyan Institute of Chemical Physics NAS RA, P. Sevak 5/2, 0014, Yerevan, Armenia, [2]Materials Research Laboratory, University of Nova Gorica, Vipavska 13, 5000, Slovenia, [3]Institute of Nano- and Biotechnologies, Aachen University of Applied Sciences, Heinrich-Mussmann-Str. 1, 52428, Jülich, Germany, [4]MicroNanoBio, Liebigstr. 4, 40479, Düsseldorf, Germany

Introduction

Urinary biomarkers are important parameters for monitoring medical conditions related to kidney disfunctions, urinary tract infection and diabetes.

A concept for an array of digital biosensors (UroLogicChip) based on electrolyte-insulator-semiconductor capacitors (EISCAP) for the point-of-care (especially, at homecare) testing of clinically relevant urinary biomarkers (urea, creatinine, glucose) is presented. The UroLogicChip will be able to provide measurement results in a YES/NO format, allowing the rapid and reliable assessment of the overal physiological condition.

Experimental results for the detection of urinary biomarker urea in phosphate buffer and artificial urine using EISCAPs modified with a stacked polyelectrolyte/enzyme urease bilayer are presented. A theoretical kinetic model of the urea-sensitive EISCAP biosensor was developed and compared with the experimentally observed results.

UroLogicChip concept

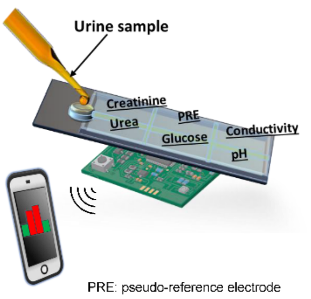

PRE: pseudo-reference electrode

- Digital display of the results in YES/NO format with cut off values for each biomarker
- Easy-to use
- Homecare, in-field settings
- Non-invasive method
- Exchangeable and combinable

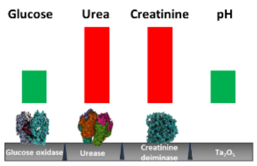

EISCAP surface modification

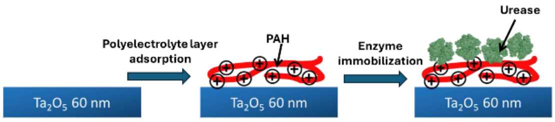

PAH: poly(allylamine) hydrochloride

Urea-sensitive EISCAP biosensor

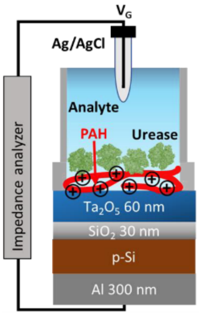

Capacitance-voltage curves

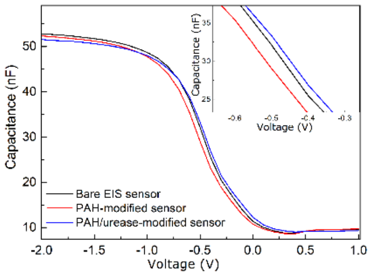

ConCap response of urea-sensitive EISCAP in buffer

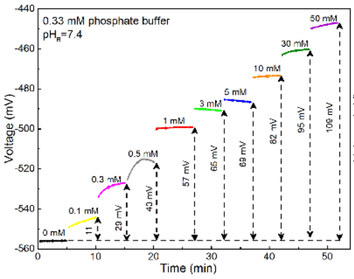

ConCap response of urea-sensitive EISCAP in artificial urine

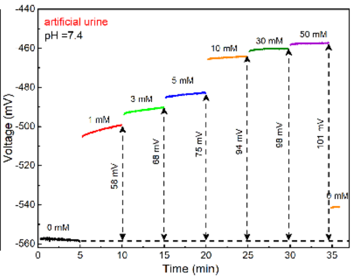

Calibration curves

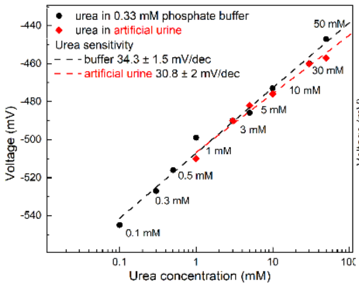

Reproducibility

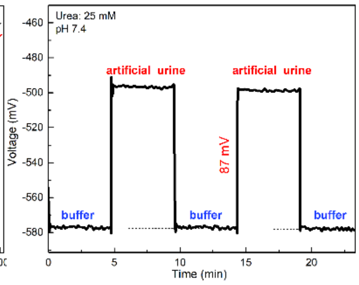

Urea/urease enzymatic reaction

$$\mathrm{NH_2{-}CO{-}NH_2(urea) + 3H_2O \xrightarrow{urease} CO_2 + 2NH_4^+ + 2OH^-}$$

Kinetic model of urea-sensitive EISCAP by Glab et al. [1]

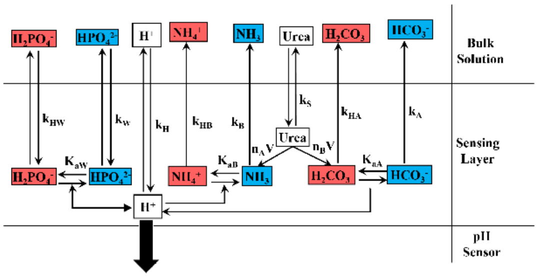

The key aspects of the kinetic model are listed below:

1. The geometry of the enzyme layer does not have to be defined in kinetic model.
2. Enzymatic reaction takes place by Michaelis-Menten kinetics, where the hydrolysis reaction rate is determined as:

$$V = \frac{V_{max}[S]}{K_m + [S]}$$

where V and **[S]** denote the actual rate and concentration of the substrate in the enzymic layer, K_m, is the Michaelis-Menten constant and V_{max} the maximum reaction rate.

3. All the species except for the enzyme can diffuse through in both directions of the enzyme layer, with transport rates proportional to the concentrations of the species.
4. The buffering components as well as the protolytic acid and base products of the enzymatic reaction are in protolytic equilibria, and the dissociation constants are the same as in the buffer solution.

Fitting of the experimental results to the kinetic model

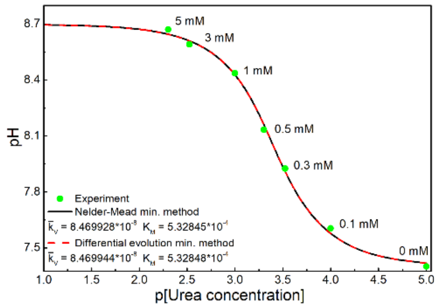

The comparison of the experimental data to this model allows to determine the relevant enzyme kinetic parameters: reaction rate (V_{MAX}) and Michaelis-Menten constant (K_m), which are of importance in the design and optimization of sensor parameters.

$\bar{k}_V$ - normalized reaction rate (V_{MAX}/K_s)

References

[1] Glab, S.; Koncki, R.; Hulanicki, A. Analyst, 116, 453 (1991).

Conclusion

The obtained results demonstrate the suitability of PAH/urease bilayer-modified EISCAPs for the urea detection in artificial urine samples with a sensitivity of 30.8 mV/dec and good reproducibility.

Acknowledgements

This research was supported by the Higher Education and Science Committee of MESCS RA (Research project № 22rl-056).
