# Supplementary material for: Urea Detection in Phosphate Buffer and Artificial Urine: A Simplified Kinetic Model of a pH-Sensitive EISCAP Urea Biosensor
Source: Sensors (Basel). 2025 Oct 26;25(21):6596. doi: 10.3390/s25216596 (PMC12608829; doi:10.3390/s25216596)
Supplement: Supplementary file 1 [file sensors-25-06596-s001.zip › SupplementaryWolframMathCode.pdf]

This supplementary Code was written for Mathematica 13.3. We are not responsible if something does not work in other versions. Make sure that the "Number of digits displayed in output" in the Wolfram Preferences are set to **15**. We encourage you to study, fix, improve, and experiment with this code in any way you want.

## Experimental data

Converting Voltage to pH. When the voltage signal becomes visually stable we take the average voltage value of 100 points of the experiment . This usually happens at the end of a long experiment, depending on your sensor and other experimental conditions.

```
ph0 =;(*starting pH*)
v0 =;(*average starting voltage, only in Volts not mV! it doesn’
 t matter if values are positive or negative*)
phs1 =;(*Your sensor's sensitivity to pH change in V/pH*)
vavg0 = {, , , ,};(*average values of voltage at steady signal
 at different substrate concentration. Again sign doesn't matter
 but make sure that first value of the list is the same as v0*)
phv[vx_, vpbs_, phs_] := ph0 + (vx - vpbs) * 1000 / phs
(*function for getting pH values from Voltage at different substrate concentration*)
```

Resulting pH should start from your v0 value and increase with each step.

```
In[ ]:= ph1 = phv[vavg0, v0, phs1](*inserting values to get pH values*)
h1 = 10^-ph1(*changing pH to H*)
```

Substrate concentration

```
sb = {, , , ,}(*your substrate concentrations in Moles/Liter(dm^3)
 number of this values should be equal to number of vavg0 values*)
sblog = -Log10[sb](*changing sb to -log10 for graphical depiction,
if you have 0 mM don't mind the ∞ symboles*)
labels = {"0 mM", "0.1 mM", "0.5 mM", "etc..."};
(*labels for every experimental dot in pH vs -lg[S concentration in mM] graph,
number of this values should be equal to number of vavg0 values*)
dots1 = N[Transpose[{sblog, ph1}]];(*table of logarithmic Sb and pH for graph*)
dots1 = ReplacePart[dots1, {1, 1} → 5](*changing the first point from ∞ to 5*)
```

If your first data point corresponds to a substrate concentration of 0 mM, replace the first element of dots1 from ∞ to 5. This adjustment is necessary because the logarithm of 0 is undefined, resulting in ∞. For example, if the smallest nonzero substrate concentration is 0.1 mM, you can safely assign the 0 mM point as $10^{-5}$ mM in the sb list.
The last command in previous block changes the first element of the table from ∞ to 5.

```
ddots1 = ListPlot[{dots1 → labels}, AxesLabel → {"Log10[Sb]", "pH"}, PlotRange → Automatic,
  BaseStyle → PointSize[0.015], PlotStyle → {Black, Red}](*Graph of experimental dots*)
```

## Constants

It is recommended to write decimal values as fractions. Because in Wolfram Mathematica decimal numbers are treated as floating-point approximations with limited precision, while fractions are stored as exact rational numbers. This way, using fractions avoids rounding errors and keeps calculations exact until a numerical approximation is explicitly requested.

```
kaA =; kaB =; kaW =;(*dissociation constants of products A,
B and buffer solution in M/L*)
nA = 1; nB = 2; (*stoichiometric coefficients of A and B products, unitless*)
kH = 1; kW = 1; (*Normalized transport rate constants accoriding to article, unitless*)
HBe = 10^-ph0;(*changing your pH to H concentration*)
cBWe =;(*buffer concentration M/L*)
```

```
nh1 = h1 / kaA(*normalization of H variable by dividing it on kaA, Ha=H/kaA*)
```

For faster calculations it is recommended to use all your CPU’s cores. By default Woflram uses single core/kernel(the first one). Other kernels are used with “Parallel...” commands. Read more about Parallelization and Kernel commands in Documentation Center and Stack Exchange.

```
Off[Solve::ratnz, NMinimize::cvmit]
ParallelEvaluate[Off[Solve::ratnz, NMinimize::cvmit]]
(*starting all your PC kernels(cpu cores) and turning off some nonimportant
 error messages, output will contain "Null" for each kernel started*)
```

## Function solving and fitting

### Solving

Simplified formula normalized according to nh1 or Ha

```
f[sB_, ha_, kV_, km_] :=
```

$$(1+ha)\left(ha+\frac{kaB}{kaA}\right)(HBe+kaW)\left(ha+\frac{kaW}{kaA}\right)\left(\left(-ha+\frac{HBe}{kaA}\right)kH+\frac{cBWe\left(-ha+\frac{HBe}{kaA}\right)kaW\,kW}{kaA^{\wedge}2*\left(ha+\frac{kaW}{kaA}\right)\left(\frac{HBe}{kaA}+\frac{kaW}{kaA}\right)}+\left(kV\left(\left(-ha-\frac{kaB}{kaA}\right)nA+ha\,(ha+1)\,nB\right)\left(km+kV-sB-\sqrt{(km+kV-sB)^{2}+4\,km\,sB}\right)\right)\Big/\left((ha+1)\,kaA\left(ha+\frac{kaB}{kaA}\right)\left(km-kV+sB+\sqrt{(km+kV-sB)^{2}+4\,km\,sB}\right)\right)\right);$$

```
(*S from Eq 6 was already inserted)*)
TraditionalForm[f[sB, ha, kV, km]](*showing result in more understandable way*)
f1[sB_, ha_, kV_, km_] = Rationalize[FullSimplify[f[sB, ha, kV, km]], 0];
(*further simplification with inserted values*)
TraditionalForm[f1[sB, ha, kV, km]]
```

Distribute all your constants/functions/variables between all started kernels. Without it kernels other than your first cannot use any of your variables. You can easily get all your definitions with command Names[“Global`*”] , copy paste this as is with all symbols inside [] brackets. Take the output of that command as input, clear quotation marks of each variable and copy/paste them in next command.

Take only those variables that are highlighted in **black**.

In[∘]:=
```
DistributeDefinitions[{cBWe, ddots1, dots1, f, f1, h1, HBe, kaA, kaB,
  kaW, kH, kW, labels, nA, nB, nh1, ph0, ph1, phs1, phv, sb, sblog, v0, vavg0}]
```

Out[∘]=
```
{cBWe, ddots1, dots1, f, kaB, kaA, HBe, kaW, kH, kW, nA,
 nB, f1, h1, labels, nh1, ph0, ph1, phs1, phv, sb, sblog, v0, vavg0}
```

Numerical solution for main equation with respect to Ha. Change Assumptions for km according to your estimates. It will take some time to solve, depending on your values, assumptions range and PC specifications it could take from 10 minutes to 1 hour or more. The result may and will look “*strange*”.

In[∘]:=
```
poly1[sB_, kV_, km_] =
 ha /. Solve[f1[sB, ha, kV, km] == 0, ha, NonNegativeReals, InverseFunctions → True,
   Assumptions → 10^-10 ≤ kV < 0.1 && 10^-10 ≤ km < 0.1 && First[sb] ≤ sB ≤ Last[sb]][[1]]
```

Example of an solution for one of our experiment in PBS. As you can see, it’s indeed “*strange*”.

Out[∘]=
```
Root[5.36355109898112 × 10^25 + 4.15525817078488 × 10^29 km +
  4.15525817078488 × 10^29 kV + 4.15525817078488 × 10^29 sB + 3.21916770200147 × 10^33 kV sB +
   (8.13176746436473 × 10^28 - 1.18985349320287 × 10^30 km - 1.18985349320287 × 10^30 kV -
     1.18985349320287 × 10^30 sB - 4.89906885672335 × 10^36 kV sB) #1 +
   (3.00134731152411 × 10^31 - 2.45666794952522 × 10^35 km - 2.45666794952522 × 10^35 kV -
     2.45666794952522 × 10^35 sB + 1.81972925431541 × 10^39 kV sB) #1^2 +
   (-6.13622780674774 × 10^32 + 2.13347301213441 × 10^35 km + 2.13347301213441 × 10^35 kV +
     2.13347301213441 × 10^35 sB + 3.35447951214354 × 10^40 kV sB) #1^3 +
   (2.36951053470875 × 10^33 + 2.07547987868224 × 10^37 km + 2.07547987868224 × 10^37 kV +
     2.07547987868224 × 10^37 sB + 2.03724248720269 × 10^41 kV sB) #1^4 +
   (6.71531742747465 × 10^33 + 5.70449467092491 × 10^37 km + 5.70449467092491 × 10^37 kV +
     5.70449467092491 × 10^37 sB + 4.70499635768171 × 10^41 kV sB) #1^5 +
   (3.71804917402002 × 10^33 + 3.68276761015288 × 10^37 km + 3.68276761015288 × 10^37 kV +
     3.68276761015288 × 10^37 sB + 3.65189247978905 × 10^41 kV sB) #1^6 +
   (1.5814151877994 × 10^31 + 7.85156883154646 × 10^34 km + 7.85156883154646 × 10^34 kV +
     7.85156883154646 × 10^34 sB) #1^7 + 1.68808729878249 × 10^28 #1^8 &, 8]
```

## Fitting

Summarizing function according to Least Squares Method Eq. (11)

In[∘]:=
```
sum1[kV_, km_] := Sum[Log10[poly1[sb[[i]], kV, km] / nh1[[i]]]^2, {i, 1, Length[nh1]}]
```

List of NMinimize Methods: "**N**elder**M**ead", "**D**ifferential**E**volution", "**S**imulated**A**nnealing", "**N**onlinear**In**teriorPoint", "**R**andom**S**earch”. Most of the time, NM and DE methods give satisfactory result. Minimizing with **DE** method takes longer time than all the others combined but the result is just slightly more accurate than all the others.

In[ ]:=
```
methods1 = {"NelderMead", "SimulatedAnnealing", "NonlinearInteriorPoint", "RandomSearch"};
(*this is a list of methods without DE to use with operators # and &/@ *)
```

```
DistributeDefinitions[poly1, sum1, methods1](*Distribute few new definitions*)
```

X stand for kV and Y for km. It is recommended that Assumptions() for X and Y here were the same as in poly1 Solve command. By default *Accuracy* and *Precision Goal*s are set to automatic which is 15, for 15 digits after the decimal point, it is **highly** not recommended to increase past this value. *MaxIterations* for **DE** method is set to 20000 which should be enough, considering how long this method takes, it is not recommended to set *Iterations* higher than this. If you want to speed up procedure you can lower *Goals* and *Iterations* values. *ParallelSubmit* automatically gets these commands ready for kernels in order starting from first. But only the next command WaitAll actually runs them. Here min1 use single kernel for all min methods except **DE**, which is set separately on next kernel with it's own *MaxIterations*.

```
min1 = ParallelSubmit[{x, y},
   NMinimize[{sum1[x, y], x ∈ PositiveReals && y ∈ PositiveReals && 10^-10 < x < 0.1 &&
        10^-10 < y < 0.1}, {x, y}, MaxIterations → 5000000, Method → #,
      AccuracyGoal → Automatic, PrecisionGoal → Automatic] & /@ methods1];
min2 = ParallelSubmit[{x, y},
   NMinimize[{sum1[x, y], x ∈ PositiveReals && y ∈ PositiveReals && 10^-10 < x < 0.1 &&
        10^-10 < y < 0.1}, {x, y}, MaxIterations → 20000, Method → #,
      AccuracyGoal → Automatic, PrecisionGoal → Automatic] & /@ methods2];
```

Output of next command will give you the list with 4 smaller list - results for each min method used and **DE** method separately, each containing 3 values: the minimum of the Sum that the command reached and small list with parameters X and Y or kV and KM.

```
res = WaitAll[{min1, min2}](*starts all minX commands in parallel.*)
```

We recommend copying the previous output and assigning it as a variable "res" separately right here. This way, you can reuse the result without rerunning the full fitting procedure after the program is reopened.
Next part of code should take the parameters and assigning them as a new variables kV(or km)(method-)(number).

```
kV = Cases[res, Rule[x, val_] :→ val, Infinity];(*collecting all results in lists*)
km = Cases[res, Rule[y, val_] :→ val, Infinity];
shortmet = {"nm", "sa", "nip", "rs", "de"};(*short names of methods,
the last result in res should be from DE*)
Do[Evaluate[Symbol["kV" <> shortmet[[i]] <> "1"]] = kV[[i]];
 Evaluate[Symbol["km" <> shortmet[[i]] <> "1"]] = km[[i]];,
 {i, Min[Length[shortmet], Length[kV], Length[km]]}](*asigning the variables*)
```

Check your variables

In[ ]:=
```
{kVnm1, kVde1, kVsa1, kVnip1, kVrs1}
{kmnm1, kmde1, kmsa1, kmnip1, kmrs1}
```

If you want to make another set of variables, copy/paste previous code and change 1 in quotation marks to other number.

Minimization methods are not guaranteed to give equally good results . In most cases, **NM** and **DE** give good results and often have similar parameters. Pay attention to “Sum” value, i.e. the first value in every method’s result, the lowest Sum value have better parameters.

### 3D visualisation of Sum kV and km

This part is optional, you can skip it.

```
In[]:= d[kV_, km_] := {kV, km, sum1[kV, km]} (*function for 3D dot*)
```

```
In[]:= mins = ListPointPlot3D[{{d[kVnm1, kmnm1]}, {d[kVde1, kmde1]},
      {d[kVsa1, kmsa1]}, {d[kVnip1, kmnip1]}, {d[kVrs1, kmrs1]}},
    PlotRange → Full, LabelStyle → Directive[Bold, Medium],
    BaseStyle → PointSize[0.03], PlotStyle → {Red, Blue, Green, Black, Purple},
    PlotLegends → {"Nelder-Mead", "Differential Evolution",
      "Simulated Annealing", "NonlinearInteriorPoint", "RandomSearch"},
    AxesLabel → {"kV", "km", "Sum"}]; (*Plotting dots in 3D*)
```

```
In[]:= min3d = Plot3D[sum1[kV, km], {kV, 0, 0.1}, {km, 0, 0.1}, PlotRange → Full, PlotPoints → 50,
    Mesh → 10, LabelStyle → Directive[Black, Bold, Medium], AxesLabel → {"kV", "km", "Sum"}];
  (*Plotting 3D surface of Sum function*)
```

You would probably need to change the variables range to find dots, change last values in {kV(or km)} brackets in *min3d* for this. You can see this range from *mins*, just delete ; and start command again to see that plot separately.

```
Show[min3d, mins] (*Showing both graphs together*)
```

## Comparing experiment with fitting

### Generation of data with newfound parameters

We set function for generating data points with fitted parameters and also convert normalized Ha back to H and to pH. These datasets can be exported to other programs to create fitting graphs.

```
ftable1[kV_, km_] := ParallelTable[
    {-Log10[sB], -Log10[poly1[sB, kV, km] * kaA]}, {sB, 10^-5, 1, 2.5 * 10^-5}];
```

```
nm1 = ftable1[kVnm1, kmnm1]; (*tables with datapoints of each method*)
de1 = ftable1[kVde1, kmde1];
sa1 = ftable1[kVsa1, kmsa1];
nip1 = ftable1[kVnip1, kmnip1];
rs1 = ftable1[kVrs1, kmrs1];
```

Turn obtained values to table of -lg[sB] and pH of every method for easy export for programs that specialize in drawing 2D graphs like Originlab

```
fited1 =
 Transpose[{nm1⟦All, 1⟧, nm1⟦All, 2⟧, de1⟦All, 2⟧, sa1⟦All, 2⟧, nip1⟦All, 2⟧, rs1⟦All, 2⟧}]
Export["fited1.csv", fited1, "CSV"]
(*By default on Windows OS this csv table is exported to your User's Documents*)
```

## Drawing graphs

```
In[ ]:= fitp1 = ListPlot[{nm1, de1, sa1, nip1, rs1}, Joined → True, PlotRange → {{1, 5.1}, {7, 9.3}},
   PlotLegends → {"Nelder-Mead", "Differential Evolution", "Simulated Annealing",
     "NonlinearInteriorPoint", "RandomSearch", "Params avg"}];

In[ ]:= Show[fitp1, ddots1]
```

# Checking for errors

Generating another smaller set of datapoints using substrate values and fitted parameters. Here we will use normalized H values, or Ha. You can delete those methods that did not gave good results, or turn them into comments.

```
nhfnm = poly1[#, kVnm1, kmnm1] & /@ sb
nhfde = poly1[#, kVde1, kmde1] & /@ sb
nhfsa = poly1[#, kVsa1, kmsa1] & /@ sb
nhfnip = poly1[#, kVnip1, kmnip1] & /@ sb
nhfrs = poly1[#, kVrs1, kmrs1] & /@ sb
nhf = {nhfnm, nhfde, nhfsa, nhfnip, nhfrs};(*list of data lists for each method*)

StandardDeviation[nh1] (*Check StandardDeviation of normalized H*)

df = Length[nh1] - 2(*Degress of Freedom,
  2 are number of fitted parameters i.e. kV and km*)

In[ ]:= mae[exp_, theor_] := Sum[Abs[exp⟦i⟧ - theor⟦i⟧], {i, 1, Length[exp]}] / Length[exp];
(*Mean absolute error*)
mse[exp_, theor_] := Sum[((exp⟦i⟧ - theor⟦i⟧)^2), {i, 1, Length[exp]}] / Length[exp];
(*Mean squarred error*)
rmse[exp_, theor_] := Sqrt[mse[exp, theor]] (*Root MSE*)
chisq1[exp_, theor_] := Sum[(exp⟦i⟧ - theor⟦i⟧)^2 / theor⟦i⟧, {i, 1, Length[exp]}];
(*Simple Chi-Squared χ² test*)
chisq2[exp_, theor_] :=
 Sum[(exp⟦i⟧ - theor⟦i⟧)^2 / StandardDeviation[theor]^2, {i, 1, Length[exp]}]
 (*Chi-Squared χ^2 test with deviation constant σ*)
```

Values are given in this order: Nelder-Mead, Differential Evolution, Simulated Annealing and Random Search

```
In[ ]:= mae[nh1, #] & /@ nhf
mse[nh1, #] & /@ nhf
rmse[nh1, #] & /@ nhf
```

```
chisq1[nh1, #] & /@ nhf (*simple χ2*)
chisq2[nh1, #] & /@ nhf (*χ2 with σ*)
chisq1[nh1, #] / df & /@ nhf (*simple normalized χ2 or just χ̄2*)
chisq2[nh1, #] / df & /@ nhf (*normalized χ̄2 with σ*)
```

Getting p – values : p = 1 – F $(\chi^2;\ df)$

F $(\chi^2;\ df)$ is CDF command

$\chi^2$ – **not** normalized chi square, or without dividing on df

df – **d**egrees of **f**reedom

The closer to 1 the better, it means there is lesser error. Again, the values are given for every min method defined in nhf

```
In[]:= pv1[nh_, nhf_] := 1 - CDF[ChiSquareDistribution[df], chisq1[nh, nhf]] (*simple χ2*)
       pv2[nh_, nhf_] := 1 - CDF[ChiSquareDistribution[df], chisq2[nh, nhf]] (*χ2 with σ*)
```

```
pv1[nh1, #] & /@ nhf
pv2[nh1, #] & /@ nhf
```

Now you can find your best kV and km pair based on lowest value of sum in fitting output, $\chi^2$ closest to 0 and/or p-value closest to 1

## Graphical fitting methods and getting the Vmax value

We assumed that transport rate constant for substrate is ks = D/(l*t) where D is diffusion constant of substrate = 9.3*10^-5 cm^2/sec, length = 1 cm and thickness 1 nm = 10^-7 cm of sensor's enzyme layer

```
ksf[l_, t_] := difc / (l * t)
difc =; (*Insert your substrates difussion constant in cm^2/sec*)
ks1 = ksf[, ] (*Insert your sensor's side length and thickness in cm *)
kVl = {kVnm1, kVde1, kVsa1, kVnip1, kVrs1}
kml = {kmnm1, kmde1, kmsa1, kmnip1, kmrs1}
vm = kVl * ks1
```

```
In[]:= fitt = TableForm[Table[{vm[[i]], kml[[i]]}, {i, 1, 5}],
         TableHeadings → {{"NM", "DE", "SA", "NIP", "RS"}, {"Vmax", "KM"}}]
```

Local substrate concentration at sensor surface. Starting from here use your best kV and km parameters. It is recommended to go back to sb and set first element from 0 to some low value like 10^-5

```
In[]:= sf[sB_, kV_, KM_] := (-KM - kV + sB + √((KM + kV - sB)^2 + 4 KM * sB)) / 2
       TraditionalForm[sf[sB, kV, km]]
       sf1[sB_] := sf[sB, kVnm1, kmnm1]
```

Out[]//TraditionalForm=

$$\frac{1}{2}\left(\sqrt{(\mathrm{km}+\mathrm{kV}-\mathrm{sB})^2+4\,\mathrm{km}\,\mathrm{sB}}-\mathrm{km}-\mathrm{kV}+\mathrm{sB}\right)$$

according to Eq. (4a) in article

```
vmax[sB_, s_, km_] := ks1 * (sB - s) (km + s) / s (*function for Vmax*)
v1[s_] := (vmax[s, sf1[s], kmnm1] * s) / (kmnm1 + s)
(*function for V values, change kmnm1 to your best one*)
TraditionalForm[vmax[sB, s, km]]
TraditionalForm[v1[sB, km]]
```

Vmax are depended on substrate concentration but it’s value changes so little that it considered a constant .

```
In[◦]:= vd1 = v1[#] & /@ sb
vsb = N[Transpose[{#, v1[#],
        vmax[#, sf1[#], kmnm1]}]] & /@ sb;
N[{TableForm[vsb, TableHeadings → {None, {"SB PBS", "V", "Vmax"}}]}]
```

## Michaelis Menten Graph

```
In[◦]:= d1 = Transpose[{sb, vd1}];
vp1 = Plot[{v1[s]}, {s, 0, 0.1}, PlotRange → Full];
vp2 = ListPlot[d1 → labels, PlotStyle → Red];
Show[vp1, vp2]
```

## Lineweaver–Burk(LB) Plot

1/V vs 1/S

```
In[◦]:= N[d1] (*Normal sb and v data*)
N[1 / d1] (*converted according to Lineweaver-Burk*)
```

```
In[◦]:= LWdata1 = {1 / #1, 1 / #2} & @@@ d1 (*setting up LB data*)
LWFit1 = LinearModelFit[LWdata1, x, x] (*Linear fitting of LB*)
```

```
In[◦]:= Show[{ListPlot[LWdata1 → labels, PlotStyle → Red, PlotMarkers → Automatic],
   Plot[LWFit1[x], {x, 0, Max[First /@ LWdata1] * 1.2}, PlotStyle → Blue]},
  PlotRange → Full, AxesLabel → {"1/[S]", "1/v"}]
```

Found Vmax and KM parameters

```
In[◦]:= LWvmax1 = 1 / LWFit1["BestFitParameters"][[1]]
LWkm1 = LWFit1["BestFitParameters"][[2]] * LWvmax1
```

## Eadie–Hofstee(EH) Plot

V vs V/S

```
In[◦]:= EHd1 = {#2 / #1, #2} & @@@ d1;
EHfit1 = LinearModelFit[EHd1, x, x];
```

```
In[◦]:= Show[{ListPlot[EHd1 → labels, PlotStyle → Red,
    PlotMarkers → Automatic, AxesLabel → {"v/[S]", "v"}],
   Plot[EHfit1[x], {x, 0, Max[First /@ EHd1] * 1.1}, PlotStyle → Blue]}, PlotRange → All]
```

```
In[•]:= EHvmax1 = EHfit1["BestFitParameters"]〚1〛
        EHkm1 = -EHfit1["BestFitParameters"]〚2〛
```

## Hanes-Woolf(HW) plot

S/V vs S

```
In[•]:= HWd1 = {#1, #1 / #2} & @@@ d1;
        HWfit1 = LinearModelFit[HWd1, x, x];

In[•]:= Show[{ListPlot[HWd1 → labels, PlotStyle → Red,
            PlotMarkers → Automatic, AxesLabel → {"[S]", "[S]/v"}],
          Plot[HWfit1[x], {x, 0, Max[First /@ HWd1] * 1.1}, PlotStyle → Blue]}, PlotRange → All]

In[•]:= HWvmax1 = 1 / HWfit1["BestFitParameters"]〚2〛
        HWkm1 = HWfit1["BestFitParameters"]〚1〛 * HWvmax1
```

Full table with fitted parameters for every graphical method

```
In[•]:= fitt = TableForm[{{vm〚1〛, kmnm1}, {LWvmax1, LWkm1}, {EHvmax1, EHkm1}, {HWvmax1, HWkm1}},
          TableHeadings → {{"Adapted Glab", "LB", "EH", "HW"}, {"Vmax", "KM"}}]
```
